# Transient Receptor Potential Melastatin 8 (TRPM8) Channel Regulates Proliferation and Migration of Breast Cancer Cells by Activating the AMPK-ULK1 Pathway to Enhance Basal Autophagy

**DOI:** 10.3389/fonc.2020.573127

**Published:** 2020-12-04

**Authors:** Yuan Huang, Shi Li, Zhenhua Jia, Weiwei Zhao, Cefan Zhou, Rui Zhang, Declan William Ali, Marek Michalak, Xing-Zhen Chen, Jingfeng Tang

**Affiliations:** ^1^ National “111” Center for Cellular Regulation and Molecular Pharmaceutics, Key Laboratory of Fermentation Engineering (Ministry of Education), Hubei University of Technology, Wuhan, China; ^2^ Membrane Protein Disease Research Group, Department of Physiology, Faculty of Medicine and Dentistry of Alberta, Edmonton, AB, Canada; ^3^ Department of Biochemistry, University of Alberta, Edmonton, AB, Canada

**Keywords:** autophagy, LC3B, transient receptor potential melastatin 8 (TRPM8), AMP-activated protein kinase(AMPK), cancer

## Abstract

The calcium-permeable cation channel TRPM8 (transient receptor potential melastatin 8) is a member of the TRP superfamily of cation channels that is upregulated in various types of cancer with high levels of autophagy, including prostate, pancreatic, breast, lung, and colon cancers. Autophagy is closely regulated by AMP-activated protein kinase (AMPK) and plays an important role in tumor growth by generating nutrients through degradation of intracellular structures. Additionally, AMPK activity is regulated by intracellular Ca^2+^ concentration. Considering that TRPM8 is a non-selective Ca^2+^-permeable cation channel and plays a key role in calcium homoeostasis, we hypothesized that TRPM8 may control AMPK activity thus modulating cellular autophagy to regulate the proliferation and migration of breast cancer cells. In this study, overexpression of TRPM8 enhanced the level of basal autophagy, whereas TRPM8 knockdown reduced the level of basal autophagy in several types of mammalian cancer cells. Moreover, the activity of the TRPM8 channel modulated the level of basal autophagy. The mechanism of regulation of autophagy by TRPM8 involves autophagy-associated signaling pathways for activation of AMPK and ULK1 and phagophore formation. Impaired AMPK abolished TRPM8-dependent regulation of autophagy. TRPM8 interacts with AMPK in a protein complex, and cytoplasmic C-terminus of TRPM8 mediates the TRPM8–AMPK interaction. Finally, basal autophagy mediates the regulatory effects of TRPM8 on the proliferation and migration of breast cancer cells. Thus, this study identifies TRPM8 as a novel regulator of basal autophagy in cancer cells acting by interacting with AMPK, which in turn activates AMPK to activate ULK1 in a coordinated cascade of TRPM8-mediated breast cancer progression.

## Introduction

The transient receptor potential melastatin 8 (TRPM8), a member of the TRP family, is a thermally regulated non-selective Ca^2+^-permeable cation channel. TRPM8 channel, also known as ‘cold receptor’, is activated by cold temperature and cooling agents (1, 2). Increasing number of studies demonstrated that TRPM8 channel is upregulated in various types of cancer, including prostate, pancreatic, breast, lung, and colon cancer (3–5) and is considered a valuable prognostic marker and putative therapeutic target (6). Advanced treatment significantly improves survival rate of patient with early-stage carcinoma; however, local recurrence or metastases after therapy failure remains common, and the mechanism of this event is incompletely understood.

Increasing evidence indicates that basal autophagy plays the critical roles in the development of local recurrence after therapy failure in human cancer (7–9). Autophagy is a highly conserved self-degradative process that functions as a cell survival mechanism during cellular stress responses, such as starvation, hypoxia, and chemo/radiotherapy (10, 11). Activation of autophagy induces the formation of autophagosomes that engulf damaged organelles or particles and fuse with lysosomes to form autophagolysosomes. Eventually, lytic enzymes within the autophagolysosomes degrade the contents of the vesicles to provide various nutrients to the cells, such as amino acids or fatty acids, which are essential for cell metabolism (12, 13). The level of basal autophagy must be properly regulated, and dysregulated autophagy is associated with neurodegeneration, microbial infection, cardiovascular diseases, muscular dystrophy, and various types of cancer (14, 15). AMP-activated protein kinase (AMPK) detects the cellular metabolic status and is considered the key regulator of basal autophagy. AMPK regulates the activity of mammalian target of rapamycin (mTOR) to control the level of autophagy. An increase in the phosphorylated form of AMPK (pAMPK) inhibits mTOR activity and leads to activation of autophagy. Moreover, an increase in the intracellular Ca^2+^ concentration leads to activation of calcium/calmodulin-dependent protein kinase kinase *β* (CaMKK*β*), which is the upstream regulator of AMPK activity that increases phosphorylation of AMPK (16, 17). Thus, intracellular Ca^2+^ concentration is an important regulator of autophagy.

Maintenance of intracellular Ca^2+^ concentration is tightly and precisely controlled by Ca^2+^ channels, pumps, and exchangers. Fluctuations in Ca^2+^ levels may contribute to cellular processes relevant to tumorigenesis, tumor progression, and autophagy, including migration, invasion, proliferation, and apoptosis (18–21). TRPM8 is a non-selective Ca^2+^-permeable cation channel, which plays the key role in calcium homoeostasis. To the best of our knowledge, whether and how TRPM8 regulates basal autophagy is unknown. In the present study, we demonstrate that TRPM8 is a potent positive regulator of basal autophagy. Moreover, TRPM8 interacts with AMPK and activates the AMPK-ULK1 signaling pathway to modulate basal autophagy.

## Materials and Methods

### Plasmids, Mutants, siRNAs, and Antibodies

The plasmids for the expression of wild type rat Trpm8 (NM_134371) and a point mutant V976W-TRPM8 in the pcDNA3 vector were obtained as described previously (22, 23). The constructs for the expression of green fluorescent protein-tagged TRPM8 (GFP-TRPM8) and Flag-tagged TRPM8 (Flag-TRPM8) were amplified by PCR using the pcDNA3-TRPM8 plasmid as a temple and subcloned into the pEGFP-N1 and pCMV10-3×Flag vectors, respectively. The constructs for the expression of Flag-tagged cytosolic domains of TRPM8 (1-691 for M8-N, 756–759 for M8-LI, 815–829 for M8-LII, and 980–1104 for M8-C) were subcloned into the pCMV10-3×Flag vector. The constructs for the expression of GST-tagged cytosolic C-terminus of TRPM8 were subcloned into the pGEX-4T-1 to express the GST-M8C fusion protein in *Escherichia coli* BL21 cells. ptfLC3 (mammalian expression of rat LC3 fused to mRFP and GFP) was a gift from Prof. Tamotsu Yoshimori (Addgene, 21074) and used to demonstrate autophagic flux by us previously (12, 24, 25). The construct for the expression of Flag-tagged PRKAA2 (protein kinase AMP-activated catalytic subunit alpha 2, AMPKa2, Flag-AMPK) was amplified by PCR using the human cDNA obtained from Pro. Jiahuai Han (Xiamen University, China) as a template and subcloned into the pCMV10-3×Flag. The siRNAs targeting human TRPM8 (siTRPM8-1: 5′-UCUCUGAGCGCACUAUUCA(dTdT)-3′ and siTRPM8-2: 5′-AGAAAUUCUCGAAUGUUCU(dTdT)-3′ were described previously) (26, 27), siRNA targeting human ATG7 (siATG7: 5′-CAGCCUGGCAUUUGAUAAA(dTdT)-3′), siRNA targeting human AMPKα1 (siAMPK: 5′-CCTCAAGCTTTTCAGGCAT(dTdT)-3′), and control siRNA (Scramble: 5′-UUCUCCGAACGUGUCACGUTT(dTdT)-3′) were synthesized by GenePharma (Suzhou, Jiangsu, China). Rabbit anti-LC3B (#18725, PTGCN, China), anti-ATG7 (#10088, PTGCN), anti-TRPM8 (#ACC-049, Alomone, Israel), anti-SQSTM1/p62 (#BM4385, Boster, China), anti-ULK1 (#20986, PTGCN), anti-phospho-ULK1 (Ser317) (#12753, Cell Signaling Technology), anti-AMPK*α*1 (#BM4202, Boster), and anti-phospho-AMPK*α* (Thr172) antibodies (#2535, Cell Signaling Technology) were used at 1:1,000 dilution. Mouse anti-GAPDH (#60004, PTGCN), anti-GFP (#66002, PTGCN), and anti-Flag (#M185, MBL) antibodies were used at 1:3,000 dilution. Goat anti-rabbit and goat anti-mouse HRP-conjugated secondary antibodies were purchased from Millipore and used at 1:20,000 dilution.

### Cell Culture and Transfection

A cervical cancer cell line (HeLa), a colorectal carcinoma cell line HCT116, breast cancer cell lines (MCF7 and MDA-MB-231), and an embryonic kidney cell line (HEK293) were used in this study. These cell lines were obtained from the Cell Center of Institute of Biochemistry and Cell Biology, Chinese Academy of Sciences (Shanghai, China) and were grown in Dulbecco’s modified Eagle’s medium (DMEM) supplemented with 10% fetal bovine serum (FBS), L-glutamine (2 mM), penicillin G (100 units/ml), and streptomycin (10 mg/ml) (Invitrogen, Merelbeke, Belgium) in a humidified incubator with 5% CO_2_ at 37°C. Cells at 70–80% confluence in a 6-well plate were transiently transfected with 2 µg of plasmid DNA or siRNA (100 pmol) per well using 5 µl of Lipofectamine 2000 according to the manufacturer’s instructions (Invitrogen). After 48 h of transfection, the cells were harvested for the assay of siRNA knockdown efficiency using western blot (WB).

To evaluate the effect of TRPM8 agonists or antagonist on cell autophagy, cells were treated for 48 h with TRPM8 agonists [10 µM menthol (Sangon Biotech, China) and 2 µM icilin (Alomone, Israel)] or a TRPM8 antagonist [0.5 µM AMTB hydrochloride (Alomone)].

### Western Blotting and Co-Immunoprecipitation

Western blotting (WB) experiments were performed using a modified protocol as described previously (28). Cells were lysed in ice-cold lysis buffer (50 mM Tris/HCl, pH 7.5, 150 mM NaCl, 2 mM EDTA, and 1% v/v NP-40) supplement with complete protease inhibitor cocktail (Roche), and the lysates were centrifuged at 13,800 × g for 10 min at 4°C. The protein extracted in the supernatant was incubated at 60°C for 5 min in 1× SDS loading buffer (6×, 0.3 M Tris/HCl, 6% SDS, 60% glycerol, 120 mM dithiothreitol (DDT) and a proprietary pink tracking dye), resolved by electrophoresis through an 8–15% SDS-polyacrylamide gel, and transferred to a PVDF membrane at 4°C. The membrane was blocked with 5% non-fat dry milk in TBST (20 mM Tris/HCl, 150 mM NaCl, and 0.05% Tween-20) for 1 h at room temperature (RT); then, the membrane was incubated with the corresponding primary antibodies overnight at 4°C. After three washes with TBST for 10 min, the membrane was incubated with a corresponding secondary antibody for 1 h at RT. The membrane was subsequently washed three times with TBST for 10 min and exposed to a SuperSignal West Pico chemiluminescent substrate (Pierce Chemical Co., Rockford, Illinois, USA). Chemiluminescence was recorded with a ChemiDoc XRS system (Bio-Rad Laboratories, Richmond, CA), and analyzed using the Image Lab software (Bio-Rad Laboratories).

Co-immunoprecipitation (Co-IP) experiments were performed using a modified protocol as described previously (29). Transfected cells were lysed in ice-cold lysis buffer for 30 min at 4°C. The supernatants of the cell lysates were precleared with 30 µl of Protein A/G PLUS-agarose (sc-2003, Santa Cruz Biotechnology) for 1 h at 4°C and centrifuged at 1,000 × g for 2 min at 4°C. Indicated antibody or corresponding control IgG (2 µg) was added with 30 µl of Protein A/G PLUS-agarose to equal amounts of precleared cell lysates, and the samples were incubated on a rotator for 2 h at 4°C; the proteins–agarose complex was centrifuged at 1,000 × g for 2 min at 4°C, washed five times with lysis buffer containing 0.1% Tween 20, and then resuspended in 50 µl of 1× SDS loading buffer (6×, 0.3 M Tris/HCl, 6% SDS, 60% glycerol, 120 mM DDT, and a proprietary pink tracking dye); the samples were incubated at 60°C for 5 min and analyzed by WB. Assays were repeated at least three times.

### Immunostaining and Confocal Microscopy

Immunostaining was performed as described previously (12). MCF7 cells grown on glass coverslips at 60% confluence were transfected with the appropriate plasmids. After 48 h of transfection, the cells were washed three times with ice-cold PBS, fixed for 10 min with 4% paraformaldehyde (w/v) in PBS, and permeabilized for 15 min by incubation with 0.5% Triton X-100 at RT. The samples were blocked with 1× PBS containing 0.1% Triton X-100 (v/v) and 10% goat serum (v/v) for 2 h and incubated with the indicated primary antibodies (*e.g.*, anti-Ki67 antibody (#27309, PTGCN) overnight at 4°C and fluorescence-labeled secondary antibodies for 2 h at RT. DAPI (1 µg/ml) was used for nuclear staining for 5 min at RT. Finally, the cells were washed three times with ice-cold PBS, and observed with a confocal laser-scanning microscopy (Leica SP8, Wetzlar, Germany). At least three fields of view were analyzed.

### Cell Proliferation and Migration Assays

Cell proliferation was determined by detecting Ki67 expression using immunostaining and by *in vitro* colony formation analysis as described previously (5, 30). For the *in vitro* colony formation assay, cells transfected with TRPM8 were plated at 1,000 cells per well in 24-well plates and cultured in the growth medium for 7–10 days; the medium was replaced every 3 days. Viable cell colonies were counted in triplicate wells using the trypan blue exclusion assay. Cell migration was determined by the wound healing assay. Briefly, cells transfected with TRPM8 constructs were seeded into 6-well plates and cultured for 24 h after the formation of a monolayer. The monolayer was scratched with a sterile 10 μl tip and washed with 1× PBS three times; the cells were cultured for 24–72 h in the conditioned medium. Cells migrating toward the wound regions were imaged and counted at 0 and 36 h time points using an inverted microscope.

### Statistical Analysis

All data are expressed as the mean ± S.E of at least three independent experiments. Statistical analysis was performed using two-tailed paired or unpaired Student’s *t* test to compare two groups. Differences were considered significant if the *P* value was < 0.05.

## Results

### TRPM8 Expression Alters Cellular Autophagy Level

**Figure 1:**
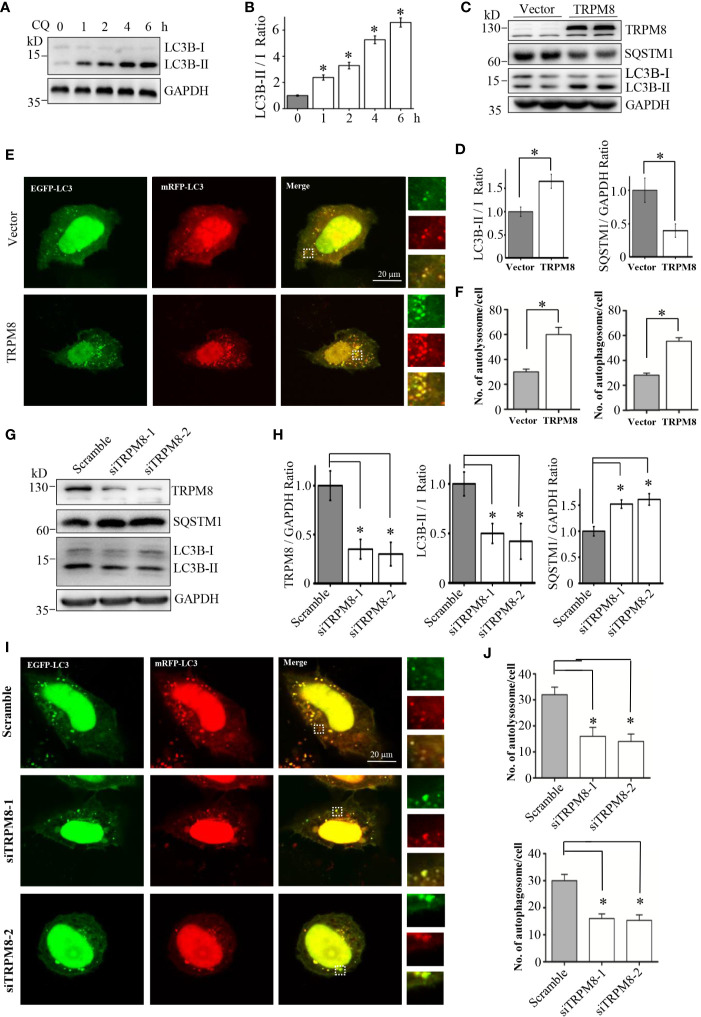
RPM8 expression activates basal autophagy level. **(A, B)** HeLa cells were incubated with 10 M CQ for the indicated times. The cell lysates were subjected to western blot (WB) analysis using indicated antibodies (N = 3). **(C, D)** Construct for TRPM8 expression was transfected into HeLa cells for 48 h; the cells were used for WB analysis using indicated antibodies (N = 3). **(E, F)** Construct for ptfLC3 expression was cotransfected with the TRPM8 or control vector into HeLa cells for 48 h, and accumulation of yellow and red puncta were observed by fluorescence microscopy; representative confocal images are shown (scale bar, 20 μm). **(G, H)** WB analysis was performed in the lysates of HeLa cells transfected with TRPM8-specific siRNA to decrease TRPM8 expression (N = 3). **(I, J)** Yellow and red puncta in HeLa cells transfected with TRPM8 siRNA. N represents the number of replicate experiments. Ctrl, control; CQ, chloroquine; LC3, microtubule-associated protein 1 light chain 3. *P < 0.05; NS, not significant.

To explore the role of TRPM8 in regulation of basal autophagy, Flag-TRPM8 was overexpressed in HeLa cells for 48 h. Then, the expression levels of SQSTM1/p62 and the conversion of LC3-I to LC3-II, which are reliable autophagy markers, were assayed. Chloroquine (CQ, Sangon Biotech, China) was used as a positive control, and CQ treatment increased the conversion of LC3-I to LC3-II thus increasing LC-II levels in HeLa cells; however, CQ also inhibits the fusion of autophagosomes with lysosomes (**Figures 1A, B**). As shown in **Figures 1C, D**, overexpression of TRPM8 significantly increased the conversion of LC3-I to LC3-II and decreased the expression level of SQSTM1/p62. To verify the effect of TRPM8 on autophagic flux, the construct for ptfLC3 expression was cotransfected with Flag-TRPM8 or a control vector into HeLa cells to observe of the differences between autophagosomes (GFP-positive/RFP-positive, yellow puncta) and autolysosomes (GFP-negative/RFP-positive, red puncta). We found that overexpression of TRPM8 markedly enhanced the generation of autophagosomes and autolysosomes with or without CQ treatment (**Figures 1E, F**). Overall, these data indicate that overexpression of TRPM8 strongly activates basal autophagy in HeLa cells.

Then, the role of endogenous TRPM8 in regulation of basal autophagy was determined. Small interfering RNA (siRNA) against human TRPM8 (siRNA-1 or siRNA-2) was transfected into HeLa cells to knockdown the endogenous expression of TRPM8. Similar to the results of previous studies (26, 27), HeLa cells transfected with two siRNAs against human TRPM8 had a significant decrease in the expression level of TRPM8 (**Figures 1G, H**). TRPM8 knockdown significantly decreased the conversion of LC3-I to LC3-II and increased the expression level of SQSTM1/p62 (**Figures 1G, H**). Confocal imaging confirmed that knockdown of TRPM8 expression significantly inhibited the generation of autophagosomes and autolysosomes with or without CQ treatment (**Figures 1I, J**). Overall, these data suggest that TRPM8 knockdown inhibits basal autophagy in HeLa cells.

### TRPM8 Channel Activity Influences Cellular Autophagy Level

**Figure 2:**
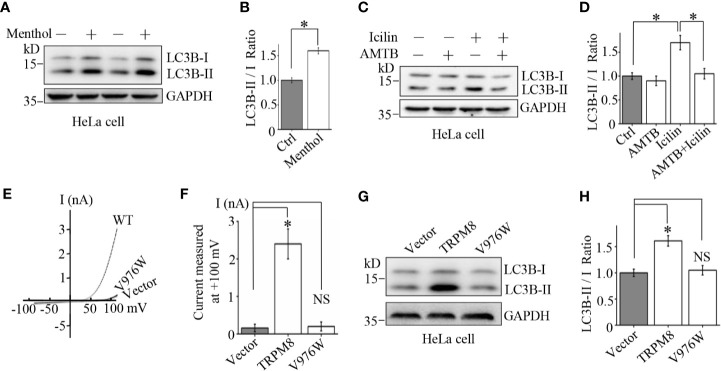
Mechanism of the stimulatory effect of TRPM8 channel function on basal autophagy. **(A, B)** HeLa cells were incubated with menthol (10 μM) for 48 h. The cells were lysed and assayed by western blot using indicated antibodies (N = 3). **(C, D)** WB analysis of HeLa cell lysates after treatment with 2 μM icilin, 0.5 μM AMTB, or their combination for 48 h (N = 3). **(E, F)** HEK293 cells were cotransfected with EGFP and wild type TRPM8, mutant, or a control vector. After 48 h of transfection, EGFP-positive cells were selected for recording of TRPM8 channel-mediated current. The number of cells used for current recordings: Vector (6); WT: (10); V976W (12). **(G, H)** HeLa cells were transfected with the constructs for the expression of wild type or mutant TRPM8. After 48 h of transfection, cells were lysed and subjected to WB analysis using indicated antibodies (N = 3). N represents the number of replicate experiments. EGFP, enhanced green fluorescent protein; Ctrl, control; *P < 0.05; NS, not significant.

The results indicated that increased expression and repression of the TRPM8 levels have opposite effects on basal autophagy in the cells. However, TRPM8 is an ion channel, and channel activity of TRPM8 may be required for the regulation of basal autophagy in the cells. To test this hypothesis, pharmacological TRPM8 agonists and an antagonist were used in combination with TRPM8 mutants to determine the role of the channel activity of TRPM8 in basal autophagy. TRPM8 channel-mediated currents are activated by cooling agents, menthol and icilin (31), and inhibited by AMTB (5). To determine the effect of TRPM8 agonists on autophagy, HeLa cells were incubated with 10 µM menthol or 2 µM icilin for 48 h. As shown in **Figures 2A–D**, menthol and icilin significantly increased the conversion of LC3-I to LC3-II. Additionally, the effect of a TRPM8 antagonist on cell autophagy was detected. AMTB is a TRPM8 channel antagonist that inhibits icilin-induced TRPM8 channel activation (5). HeLa cells were treated with 2 µM icilin, 0.5 µM AMTB, or their combination for 48 h. As shown in **Figures 2C, D**, treatment with AMTB suppresses icilin-induced increase in the conversion of LC3-I to LC3-II. Thus, channel activity of TRPM8 regulated by pharmacological agents plays an important role in cellular autophagy.

To confirm the role of TRPM8 channel activity on cellular autophagy, a mutant V976W-TRPM8 mutant in the gate region was used. Similar to our previous report (22), the mutant V976W-TRPM8 mutant did not produce robust currents at negative voltages unlike wild type TRPM8 in HEK293 cells (**Figures 2E, F**). Consistently, the mutant V976W-TRPM8 mutant failed to increase the conversion of LC3-I to LC3-II in HeLa cells (**Figures 2G, H**). Overall, these data provide a proof-of-concept that TRPM8 channel activity plays a critical role in the regulation of cellular autophagy.

### TRPM8 Regulates Cellular Autophagy in Other Cancer Cell Lines

**Figure 3:**
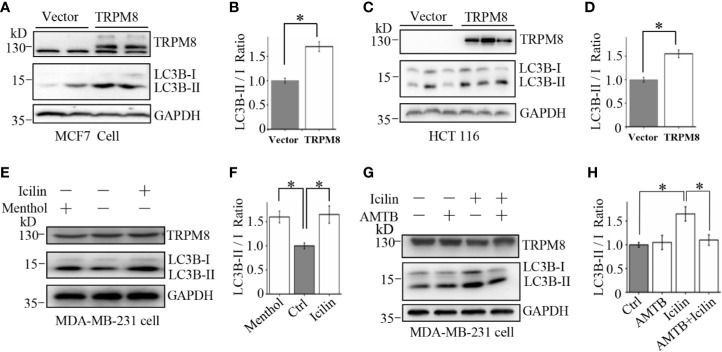
Upregulation of autophagy by TRPM8 in various cancer cell lines. **(A, B)** The construct for TRPM8 expression was transiently transfected into MCF7 for 48 h; the cells were used for WB analysis using indicated antibodies to detect autophagy-associated proteins (N = 3). **(C, D)** Similar WB analysis of lysates of HCT116 cells transfected with TRPM8 (N = 3). **(E–H)** WB analysis of lysates of MDA-MB-231 cells treated with 10 μM menthol, 2 μM icilin, 0.5 μM AMTB, or combined treatments for 48 h (N = 3). N represents the number of replicate experiments. *P < 0.05; NS, not significant.

To determine whether TRPM8-activated autophagy is specific to HeLa cells, similar experiments were performed in other types of cells. An increase in the expression of TRPM8 significantly increased the conversion of LC3-I to LC3-II and activated basal level of autophagy in adenocarcinoma MCF7 cells and colorectal carcinoma HCT116 cells (**Figures 3A–D**). Additionally, treatment with menthol or icilin enhanced the conversion of LC3-I to LC3-II in breast cancer MDA-MB-231 cells to the levels similar to those detected in HeLa cells, whereas treatment with AMTB suppressed icilin-induced increase in the conversion of LC3-I to LC3-II in MDA-MB-231 cells (**Figures 3E–H**). Thus, these data indicate that TRPM8 promotes basal autophagy in several types of mammalian cancer cells suggesting that TRPM8 is a universal master regulator of basal autophagy.

### TRPM8–AMPK Interaction Leads to AMPK Activation to Enhance Cellular Autophagy

**Figure 4:**
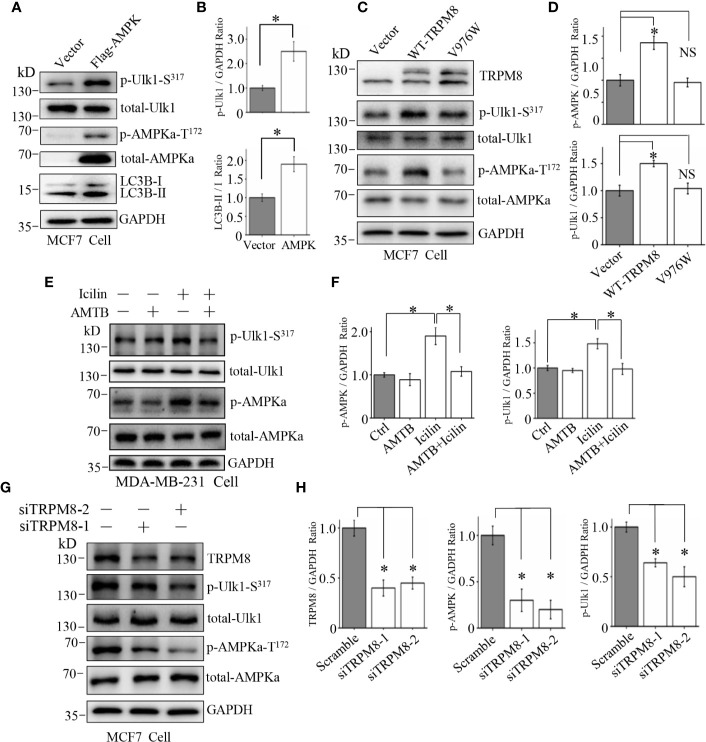
Involvement of the AMPK-ULK1-LC3 signaling cascade in TRPM8-stimulated autophagy. **(A, B)** The construct for Flag-AMPK expression was transiently transfected into MCF7 cells. After 48 h of transfection, the AMPK-ULK1-LC3 signaling cascade-related proteins were detected by WB (N = 3). **(C, D)** MCF7 cells were transiently transfected with wild type TRPM8, mutant V976W, or control vector. After 48 h of transfection, protein lysates were used for WB analysis (N = 3). **(E, F)** MDA-MB-231 cells treated with 2 μM icilin, 0.5 μM AMTB, or their combination for 48 h were extracted for WB analysis (N = 3). **(G, H)** MCF7 cells were transfected with siRNA against human TRPM8; siRNA against TRPM8 (siTRPM8-1 and siTRPM8-2) successfully knocked down TRPM8 expression compared with that in the control scramble siRNA samples according to WB analysis using an anti-TRPM8 antibody. The AMPK-ULK1-LC3 signaling cascade-related proteins detected by WB analysis using the indicated antibodies (N = 3). N represents the number of replicate experiments. *P < 0.05; NS, not significant.

TRPM8 is a calcium-permeable non-selective cation channel of the transient receptor potential superfamily (32). Recently, [Ca^2+^]_i_ has been reported to regulate autophagy by influencing phosphorylation of AMPK, which is the key regulator of basal autophagy that phosphorylates and activates ULK1 during the initiation of autophagy (33). Thus, it is essential to determine whether TRPM8 regulates basal autophagy *via* the AMPK-ULK1 signaling pathway. MCF7 cells were transiently transfected with Flag-TRPM8 for 48 h. As shown in **Figures 4A, B**, MCF7 cells transfected with Flag-AMPK had significantly activated basal autophagy and increased level of ULK1 phosphorylation as expected for a positive control. Overexpression of TRPM8 increased the levels of AMPK and ULK1 phosphorylation (pAMPK and pULK1, respectively), whereas the total expression levels of AMPK and ULK1 were unchanged (**Figures 4C, D**), suggesting that TRPM8 activates the AMPK-ULK1 signaling pathway. However, the mutant V976W-TRPM8 mutant did not activate the AMPK-ULK1 signaling pathway (**Figures 4C, D**) in agreement with the data that V976W-TRPM8 does not produce robust currents at negative voltages in electrophysiological assays (**Figures 2E, F**). Additionally, MDA-MB-231 cells were treated with 2 µM icilin, 0.5 µM AMTB, or their combination for 48 h. Cells treatment with a TRPM8 agonist icilin activated the AMPK-ULK1 signaling pathway by increasing the levels of pAMPK and pULK1, whereas an antagonist AMTB inhibited icilin activation of the AMPK-ULK1 signaling pathway (**Figures 4E, F**). Moreover, TRPM8 knockdown inhibited the AMPK-ULK1 signaling pathway in MCF7 cells (**Figures 4G, H**). These data indicate that TRPM8 is a positive regulator activating the AMPK-ULK1 signaling pathway, suggesting that TRPM8 regulation of basal autophagy may be mediated by the AMPK-ULK1 signaling pathway.

**Figure 5:**
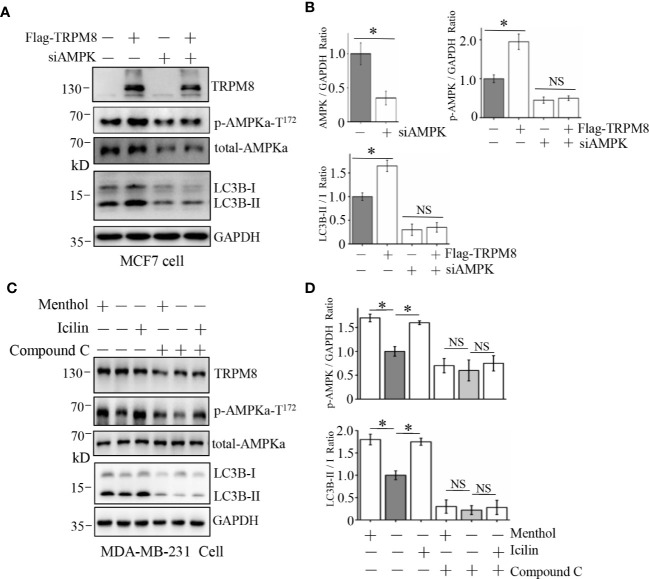
Influence of AMPK impairment on the stimulatory effect of TRPM8 on autophagy. **(A, B)** MCF7 cells were transiently transfected with siRNA against human AMPK and a Flag-TRPM8 construct. After 48 h of transfection, protein lysates were extracted for WB analysis to determine the effect of TRPM8 overexpression on basal autophagy in the presence of AMPK knockdown (N = 3). **(C, D)** WB analysis of cell lysates of MDA-MB-231 cells treatment with 2 μM icilin, 10 μM menthol, or a combination of 10 M compound C for 48 h (N = 3). N represents the number of replicate experiments. *P < 0.05; NS, not significant.

To confirm this suggestion, siRNA against human AMPK and compound C (Selleck), a specific blocker of AMPK, were used to decrease AMPK expression and suppress the AMPK kinase activity, respectively. As shown in **Figures 5A, B**, siRNA against human AMPK was transfected into MCF7 cells to successfully knockdown the endogenous expression of AMPK. Furthermore, AMPK knockdown impaired the stimulatory effect of TRPM8 overexpression on the conversion of LC3-I to LC3-II, suggesting that AMPK expression is required for regulation of basal autophagy by TRPM8. Then, AMPK activity was inhibited, and positive regulation of basal autophagy by TRPM8 was assayed. As shown in **Figures 5C, D**, treatment of MDA-MB-231 cells with 10 µM compound C inhibited AMPK phosphorylation by blocking AMPK activation as expected and inhibited ULK1 phosphorylation. Moreover, compound C impaired the stimulatory effect of TRPM8 activation by menthol or icilin on basal autophagy level, suggesting that TRPM8 regulation of basal autophagy is dependent on AMPK activity. Thus, these data indicate that impaired AMPK activity or expression suppress the regulatory effect of TRPM8 on basal autophagy.

**Figure 6:**
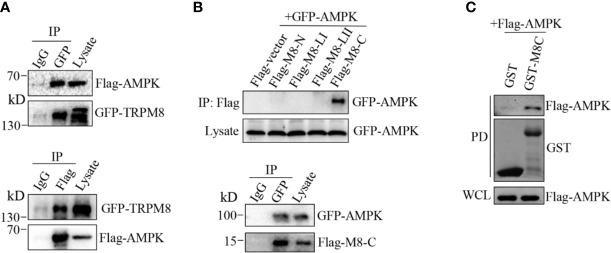
AMPK interacts with TRPM8. **(A, B)** Co-IP analysis. **(A)** Constructs for GFP-TRPM8 and Flag-AMPK expression were transiently transfected into MCF7 cells. After 48 h of transfection, protein lysates were immunoprecipitated with an anti-GFP antibody and assayed by immunoblot with an anti-Flag antibody (lower panel). Reciprocal Co-IP with an anti-Flag antibody used for immunoprecipitation and anti-GFP used for WB analysis (upper panel) (N = 3). **(B)** The construct for GFP-AMPK expression was cotransfected with M8-N, M8-LI, M8-LII, or M8-C into MCF7 cells. Protein lysates were immunoprecipitated with an anti-Flag antibody and assayed by immunoblot with an anti-GFP antibody (upper). The constructs for GFP-AMPK and Flag-M8-C expression were cotransfected into MCF7 cells. Protein lysates were immunoprecipitated with an anti-GFP antibody and assayed by immunoblot with an anti-Flag antibody (upper) (N = 3). **(C)** GST pull-down analysis. Protein lysates of MCF7 cells transiently expressing Flag-AMPK were incubated with purified cytoplasmic C-terminus of TRPM8 GST fusion protein (GST-M8C). GST-M8C, but not control GST, successfully pulled down Flag-AMPK. PD: pull-down. The lysate was used as a positive control (N = 3). N represents the number of replicate experiments. PD, pull down; WCL, whole cell lysates.

AMPK interacts with ion channels, including sodium channel Na_V_1.5 and the cystic fibrosis transmembrane conductance regulator (CFTR), forming a protein complex (34–37). Thus, we aimed to determine whether TRPM8 directly interacts with AMPK to promote AMPK activation and phosphorylation. Co-IP was performed using protein extracts from MCF7 cells cotransfected with constructs for the expression of GFP-TRPM8 and Flag-AMPK. Precipitation with anti-GFP antibody resulted in pull-down of TRPM8 and AMPK, reciprocally, anti-Flag antibody was able to pull-down AMPK and TRPM8 (**Figure 6A**). Additionally, the role of the cytoplasmic region of TRPM8 in the interaction with AMPK was determined; the data of the reciprocal Co-IP experiments indicated that cytoplasmic C-terminus of TRPM8 (M8C) interacts with AMPK *in vivo* (**Figure 6B**). The results of the GST pull-down assays *in vitro* further confirmed the interaction of AMPK with TRPM8. GST-M8C purified from *Escherichia coli* BL21 cells effectively pulled down AMPK expressed in MCF7 cells, which was not pulled down with GST (**Figure 6C**); thus, TRPM8 specifically binds AMPK. These data suggest that TRPM8 interacts with AMPK in a protein complex, and this interaction is mediated by the cytoplasmic C-terminus of TRPM8. Thus, TRPM8 regulates AMPK function *via* TRPM8**–**AMPK interaction that stimulates phosphorylation and activation of AMPK, which in turn promotes ULK1 activation to enhance basal autophagy.

### Autophagy Mediates the Regulatory Effects of TRPM8 on the Proliferation and Migration of Breast Cancer Cells

**Figure 7:**
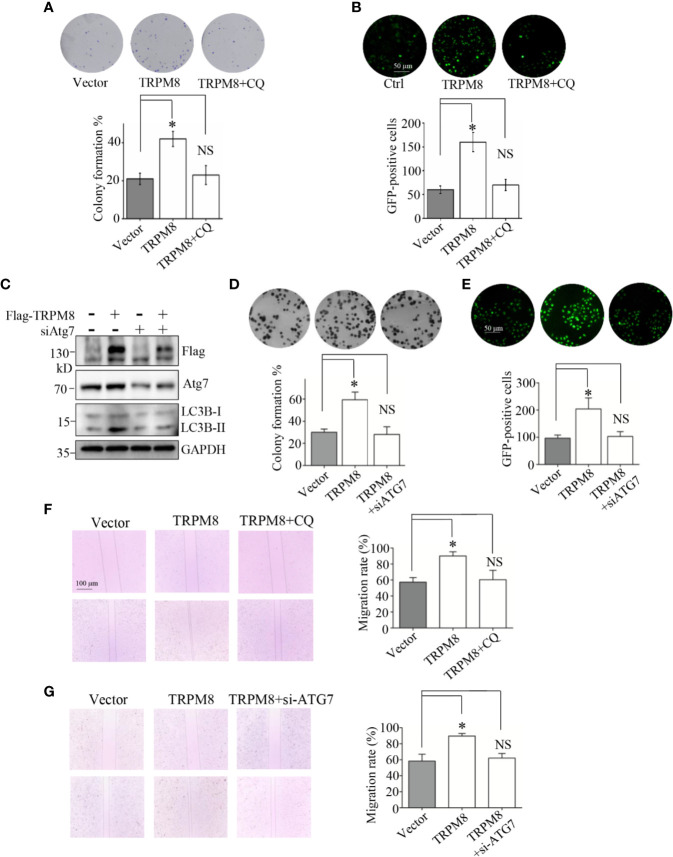
Autophagy is essential for the regulatory effect of TRPM8 on the proliferation and migration of breast cancer cells. **(A, B)** Cell proliferation experiments. MCF7 cells were transfected with TRPM8 constructs. After 24 h of transfection, cells were treated with 10 M CQ. **(A)** The *in vitro* colony formation assay. Cells were further cultured in growth media for 7-10 days to form the colonies. The colonies were washed with ice-cold PBS three times, stained with trypan blue, and counted (N = 3). **(B)** Ki67 expression was detected by immunostaining (N = 3). **(C)** MDA-MB-231 cells were transiently transfected with siRNA against human ATG7 (siATG7) and a Flag-TRPM8 construct. After 48 h of transfection, protein lysates were extracted for WB analysis to determine the effect of TRPM8 overexpression on basal autophagy in the presence of ATG7 knockdown (N =3). **(D, E)** Similar experiment was performed in MDA-MB-231 cells transfected with siATG7 and a Flag-TRPM8 construct. (N = 3). **(F, G)** Cell migration determined by wound healing assay. **(F)** MCF7 cells were transfected with the TRPM8 constructs for 24 h and scratched with a sterile 10 μl tip. After three washes with 1× PBS, the cells were cultured for 24–72 h in serum-free medium in the presence of 10 μM CQ (N = 3). **(G)** Similar wound healing assay as in **(F)** but with MDA-MB-231 cells transfected with siATG7 and a Flag-TRPM8 construct (N = 3). N represents the number of replicate experiments. *P < 0.05; NS, not significant.

Previous studies showed that TRPM8 is significantly upregulated in several types of cancer cells, including breast cancer cells, and promotes their proliferation and migration (3–5). Autophagy has opposing, context-dependent roles in cancer and impacts the development of local recurrence after therapy failure (7–9, 13); thus, autophagy may mediate the regulation of proliferation and migration by TRPM8 in breast cancer cells. The effect of TRPM8 on the colony formation was determined. As shown in **Figure 7A**, the *in vitro* colony formation of TRPM8-transfected MCF7 cells was significantly enhanced compared to that in the control vector-transfected cells similar to the results of a previous study in TRPM8-transfected PC-3 or LNCaP cells (5, 38). However, CQ, an autophagy inhibitor, abolished TRPM8-dependent enhancement of colony formation (**Figure 7A**). To verify the role of autophagy in the effects of TRPM8 on the regulation of growth of breast cancer cells, the proliferation marker Ki67 was assayed. As shown in **Figure 7B**, overexpression of TRPM8 markedly enhanced Ki67 expression in MCF7 cells, whereas CQ reversed this effect. ATG7, an essential regulator of autophagy, involved in the cleavage of LC3-I to LC3-II and the subsequent incorporation of LC3-II into the autophagosome membrane (39, 40). To provide further documentation, siRNA against human ATG7 (siATG7) was used to knockdown ATG7 expression. ATG7 knockdown impaired the stimulatory effect of TRPM8 overexpression on the conversion of LC3-I to LC3-II (**Figure 7C**). Moreover, ATG7 knockdown abolished TRPM8-dependent increase in colony formation and Ki67 expression in MDA-MB-231 cells (**Figures 7D, E**). These results suggest that autophagy is involved in the regulation of malignant growth of breast cancer cells *in vitro* by TRPM8. Previous studies showed that TRPM8 enhances breast cancer cell migration by wound-healing assays (4). In the present study, TRPM8 overexpression promoted the migration of MCF7 cells according to the data of the wound-healing assays (**Figure 7F**). However, TRPM8 failed to increase the migration of MCF7 cells in the presence of CQ (**Figure 7F**). Additionally, ATG7 knockdown impaired an increase in the migration of MDA-MB-231 cells transfected with TRPM8 (**Figure 7G**), suggesting that autophagy mediates the effect of TRPM8 on the migration of breast cancer cells. Overall, autophagy is involved in TRPM8-depedent regulation of the proliferation and migration of breast cancer cells.

## Discussion

TRPM8 is a thermally regulated non-selective Ca^2+^-permeable cation channel. Previous studies demonstrated that cytosolic Ca^2+^ is an important regulator of autophagy (16, 17); however, it is not known whether TRPM8 is involved in the regulation of basal autophagy. This study demonstrated that TRPM8 as a positive regulator that promotes basal autophagy in several types of mammalian cancer cells. Furthermore, TRPM8 interacts with and activates AMPK to promote ULK1 phosphorylation to enhance basal autophagy.

AMPK is an evolutionarily conserved serine/threonine protein kinase that is the key energy sensor and regulator of cellular metabolism to maintain energy homeostasis in eukaryotic cells. AMPK is a heterotrimeric complex composed of a catalytic α-subunit and two regulatory subunits, *β* and *γ*. The *α*-subunit contains the kinase domain and a critical Thr172 residue phosphorylated by an upstream kinase. Activated AMPK regulates a variety of metabolic processes, including autophagy. AMPK directly promotes autophagy by phosphorylating Ser317 and Ser777 of ULK1 to activate ULK1 and induce autophagy initiation by a coordinated cascade (41, 42). The present study demonstrated that TRPM8 regulation of basal autophagy is dependent on AMPK and that TRPM8 positively regulates AMPK activity to stimulate the AMPK-ULK1 signaling pathway. Thus, our study expands the list of the upstream regulatory factors of AMPK. Previous studies showed that AMPK interacts with and phosphorylated CFTR to negatively regulate CFTR function (34, 36, 37). Additionally, AMPK interacts with and phosphorylates Na_V_1.5 to regulate the interaction of Na_V_1.5 with LC3 that is important for autophagic degradation (35). Considering that AMPK interacts with TRPM8 *via* the cytoplasmic C-terminus in a protein complex, additional studies are needed to determine whether and how AMPK phosphorylates TRPM8 at the C-terminus to modulate TRPM8 function.

TRP channels precisely regulate intracellular Ca^2+^ concentrations ([Ca^2+^]_i_), which influence AMPK activity. Thus, TRP channels, such as TRPML1, TRPML2, TRPML3, TRPV1, TRPC1, TRPM2, TRPM3, and TRPM7, are involved in the regulation of autophagy. However, these TRP channels have opposite effects on autophagy suggesting that Ca^2+^ regulation of autophagy is complex (43). Similar to the TRPM (melastatin) subfamily, *i.e.*, TRPM2, TRPM3, and TRPM7 (33, 44, 45), TRPM8 positively regulates basal autophagy according to the results of the present study, which extend our understanding of the effects of the TRPM subfamily on autophagy. However, it is not known whether and how the other TRPM subfamily members, *i.e.*, TRPM1, TRPM4, TRPM5, and TRPM6, are involved in the regulation. Additionally, similar to TRPM3 and TRPM7, TRPM8 may be phosphorylated by AMPK to induce TRPM8 activation to regulate autophagy. TRPM8 lacks a kinase domain; hence, additional experiments are needed to identify protein kinases involved in the regulation of AMPK phosphorylation by TRPM8 through the TRPM**–**AMPK interaction.

TRPM8 channel is a Ca^2+^-permeable cation channel that belongs to the TRPM subfamily of TRP proteins. TRPM8 is activated by cold temperature and menthol and is also known as a ‘cold receptor’ (5). Increasing evidence indicates that the abnormal expression of TRPM8 is associated with various types of cancer, including pancreatic, prostate, breast, lung, and colon cancer and osteosarcoma; TRPM8 channels contribute to tumor growth and metastasis (3–5). The mechanism of stimulation of tumor growth and metastasis by TRPM8 was actively investigated. For example, TRPM8 was shown to activate the AKT–GST-3*β* pathway and regulate epithelial–mesenchymal transition (EMT) that promotes breast cancer metastasis (4). Additionally, TRPM8 reduces RACK1-mediated ubiquitination of HIF-1*α* to elevate HIF-1*α* expression during the hypoxic growth adaptation of prostate cancer cells (5). Local recurrence or metastases after therapy failure in patients with early-stage carcinoma are common. Emerging evidence indicates that autophagy plays the critical roles in the development of local recurrence after therapy failure in human cancers (7–9). Our findings indicate that TRPM8 is a universal master regulator of basal autophagy that maintains and regulates the level of basal autophagy in several types of mammalian cancer cells. Furthermore, the regulatory effects of TRPM8 on the proliferation and migration of breast cancer cells are dependent on autophagy. Since upregulation of TRPM8 channel is implicated in several autophagy-related cancers, the regulation of basal autophagy by TRPM8 channel may be a possible therapeutic target.

## Data Availability Statement

The original contributions presented in the study are included in the article/supplementary material. Further inquiries can be directed to the corresponding author.

## Author Contributions

YH, SL, ZJ, and WZ performed experiments. YH, CZ, RZ, DA, MM, X-ZC, and JT analyzed data. YH, and JT wrote the manuscript. YH, X-ZC, and JT conceived and designed the study. All authors contributed to the article and approved the submitted version.

## Funding

This study was supported by the China National Science Foundation (grants 31871420 to JT and 31701228 to CZ), the Hubei Province Science Foundation (grant 2019CFB166 to YH), the Wuhan Science and Technology Project (2019020701011475 to JT) and the Doctoral Scientific Research Foundation of Hubei University of Technology (grants BSQD2017032 to YH).

## Conflict of Interests

The authors declare that the research was conducted in the absence of any commercial or financial relationships that could be construed as a potential conflict of interest.
